# Serum amyloid-beta levels are increased in patients with obstructive sleep apnea syndrome

**DOI:** 10.1038/srep13917

**Published:** 2015-09-09

**Authors:** Xian-Le Bu, Yu-Hui Liu, Qing-Hua Wang, Shu-Sheng Jiao, Fan Zeng, Xiu-Qing Yao, Dong Gao, Ji-Chuan Chen, Yan-Jiang Wang

**Affiliations:** 1 Department of Neurology and Center for Clinical Neuroscience, Daping Hospital, Third Military Medical University, Chongqing 400042, China; 2 Department of Sleep center, Daping Hospital, Third Military Medical University, Chongqing 400042, China; 3 Department of Otolaryngology Head and Neck Surgery, Daping Hospital, Third Military Medical University, Chongqing 400042, China

## Abstract

A critical link between amyloid-beta (Aβ) and hypoxia has been demonstrated in *in vitro* and animal studies but has not yet been proven in humans. Obstructive sleep apnea syndrome (OSAS) is a common disorder that is characterized by nocturnal intermittent hypoxaemia. This study sought to examine the association between the chronic intermittent hypoxia and Aβ in OSAS patients. Forty-five cognitively normal OSAS patients and forty-nine age- and gender-matched subjects diagnosed with simple snoring and not OSAS were included in the present study. Serum Aβ40, Aβ42, total tau and phosphorylated tau 181 (P-tau 181) levels were measured using ELISA kits. All subjects were evaluated with nighttime polysomnography and cognitive tests. Compared with the controls, the OSAS patients exhibited significantly higher serum Aβ40, Aβ42 and total Aβ levels, and each of these levels was positively correlated with the apnea-hypopnea index, the oxygen desaturation index, and the mean and lowest oxyhaemoglobin saturations in the OSAS patients. Moreover, the OSAS patients exhibited strikingly higher serum P-tau 181 levels, and these levels were positively correlated with serum Aβ levels. This study suggests that there is an association between chronic intermittent hypoxia and increased Aβ levels, implying that hypoxia may contribute to the pathogenesis of Alzheimer’s disease.

Alzheimer’s disease (AD) is the most common age-related neurodegenerative disorder and is characterized by progressive memory loss and cognitive decline. Senile plaques consisting of abnormally aggregated amyloid-beta (Aβ) peptide are a major pathological hallmark of AD. Aβ has been suggested to play a pivotal role in the pathogenesis of this devastating disease^1^. Many environmental factors are involved in the aetiology of AD, and hypoxia is increasingly recognized as being associated with an increased risk of AD^2^. Recent data have shown that hypoxia is involved in the metabolism of Aβ in animal and cellular models^3^,^4^,^5^. However, the effect of hypoxia on Aβ metabolism in humans remains unknown. Obstructive sleep apnea syndrome (OSAS) is a prevalent disorder that is characterized by nocturnal intermittent hypoxaemia that results from episodes of partial or complete upper airway obstruction during sleep^6^. Thus, OSAS can be viewed as a chronic intermittent hypoxia model in humans. In the present study, we aimed to investigate whether Aβ and phosphorylated tau (P-tau) levels are associated with hypoxia in OSAS patients.

## Results

### Characteristics of the study population

As shown in Table 1, 45 OSAS patients (14 patients with mild, 13 with moderate and 18 with severe OSAS) and 49 age- and gender-matched subjects with simple snoring were included in this study. The controls and OSAS patients were similar regarding educational level (*p* = 0.595), smoking history (*p* = 0.834) and body mass index (BMI) (*p* = 0.146). There were no significant differences in the comorbidities of hypertension, diabetes mellitus, cardiovascular disease or hyperlipidaemia between the controls and the OSAS group. By definition, the OSAS patients exhibited higher apnea-hypopnea index (AHI) (*p* < 0.001), arousal index (ArI) (*p* < 0.001) and oxygen desaturation index (ODI) (*p* < 0.001) than the controls, and the mean oxyhaemoglobin saturation (MSaO_2_) (*p* < 0.001) and the lowest oxyhaemoglobin saturation (LSaO_2_) (*p* < 0.001) were dramatically lower in the OSAS group. Neither total sleep time (TST) nor sleep efficiency was significantly different between the two groups. The OSAS patients exhibited greater proportions of stages 1 and 2 sleep (*p* = 0.003), and stage 3 sleep (*p* = 0.03) and rapid eye movement (REM) sleep (*p* = 0.006) occupied lower proportions of the sleep time of the OSAS patients.

### Serum Aβ levels in the controls and OSAS patients

**Figure 1:**
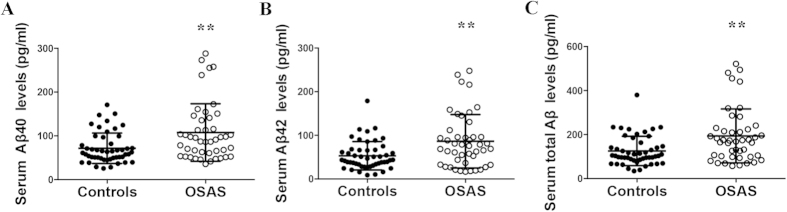
Comparison of the serum Aβ levels between the controls and patients with OSAS. The Aβ40 (**A**), Aβ42 (**B**) and total Aβ (**C**) levels were increased in the patients with OSAS. ^**^denotes *p* < 0.01.

As presented in Fig. 1, the serum Aβ40 (107.54 ± 65.77 pg/ml vs. 71.58 ± 34.70 pg/ml, *p* = 0.003), Aβ42 (86.37 ± 61.14 pg/ml vs. 50.91 ± 26.93 pg/ml, *p* = 0.006) and total Aβ (193.91 ± 122.41 pg/ml vs. 125.35 ± 66.67 pg/ml, *p* = 0.002) levels were significant higher in the OSAS patients than in the controls.

### Correlations of serum Aβ levels with AHI, MSaO_2_, LSaO_2_ and ODI in the OSAS patients

**Figure 2:**
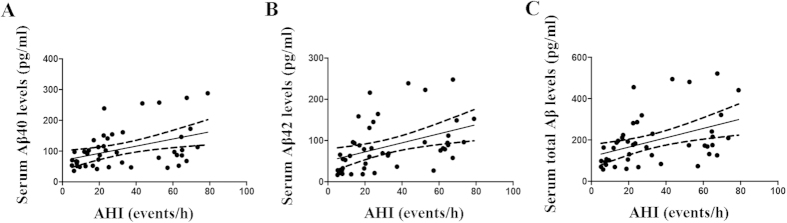
Correlations between the serum Aβ levels and AHI in the patients with OSAS. (**A**) The Aβ40 (r = 0.333, p = 0.029), (**B**) Aβ42 (r = 0.313, p = 0.041) and (**C**) total Aβ (r = 0.336, p = 0.027) levels in the serum were positively correlated with the AHI in the OSAS patients.

**Figure 3:**
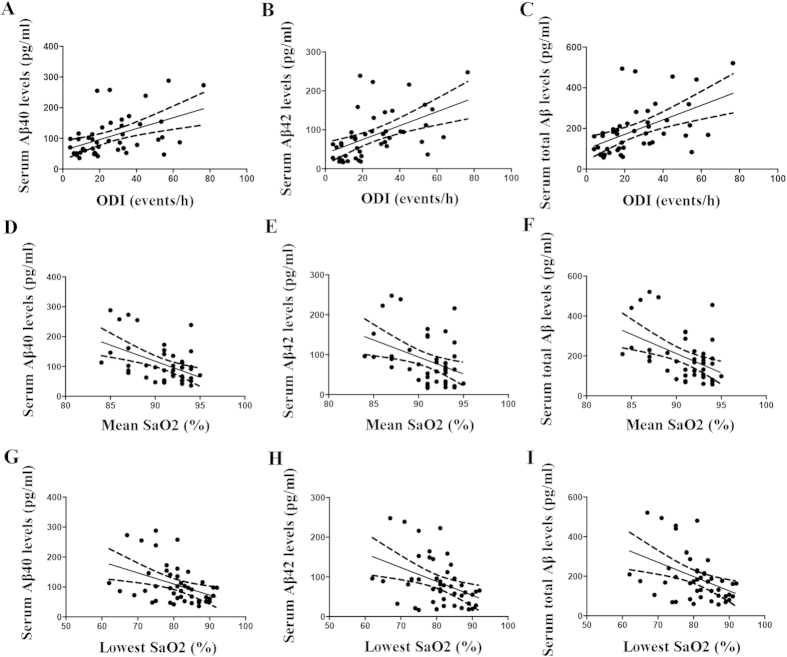
Correlations of the serum Aβ levels with the ODI, mean SaO_2_ and lowest SaO_2_ in the patients with OSAS. (**A**–**C**) show the positive correlations of the Aβ40 (r = 0.507, p = 0.003), Aβ42 (r = 0.517, p = 0.002) and total Aβ (r = 0.529, p = 0.002) levels with the ODI. (**D**–**F**) show the negative correlations of the Aβ40 (r = −0.429, p = 0.011), Aβ42 (r = −0.358, p = 0.038) and total Aβ (r = −0.408, p = 0.017) levels with the mean SaO_2_. (**G**–**I**) show the negative correlations of the Aβ40 (r = −0.339, p = 0.050), Aβ42 (r = −0.303, p = 0.082) and total Aβ (r = −0.332, p = 0.055) levels with the lowest SaO_2_.

Partial correlation analyses were used to investigate the correlations of serum Aβ levels with AHI with adjustments for age, gender, educational level, smoking history, BMI and comorbidities. In the model that included all of the participants, the serum Aβ levels were positively correlated with AHI (Supplementary Table 1). In the model that included only the OSAS patients, the serum Aβ40 (*r* = 0.333, *p* = 0.029), Aβ42 (*r* = 0.313, *p* = 0.041) and total Aβ (*r* = 0.336, *p* = 0.027) levels were also positively correlated with the AHI (Fig. 2). Next, we analysed the associations of the serum Aβ levels with the extent of hypoxia while adjusting for age, gender, educational level, smoking history, BMI, comorbidities and sleep quality. We found that the serum Aβ level was negatively correlated with MSaO_2_ and LSaO_2_ and positively correlated with ODI in the model that included all of the subjects (Supplementary Table 1). In the model that included only the OSAS patients, the serum Aβ40 (*r* = 0.507, *p* = 0.003), Aβ42 (*r* = 0.517, *p* = 0.002) and total Aβ(*r* = 0.529, *p* = 0.002) levels were all positively correlated with ODI (Fig. 3A–C). Additionally, the serum Aβ40 (*r* = −0.429, *p* = 0.011), Aβ42 (*r* = −0.358, *p* = 0.038) and total Aβ levels (*r* = −0.408, *p* = 0.017) were all negatively correlated with MSaO_2_ (Fig. 3D–F), and there was no significant correlation between the serum Aβ level and LSaO_2_ (Aβ40: *r* = −0.339, *p* = 0.050; Aβ42: *r* = −0.303, *p* = 0.082; Total Aβ: *r* = −0.332, *p* = 0.055; Fig. 3G–I). These data indicated that the Aβ levels were closely related to the extent of hypoxia.

### Serum tau protein levels in the controls and OSAS patients

**Figure 4:**
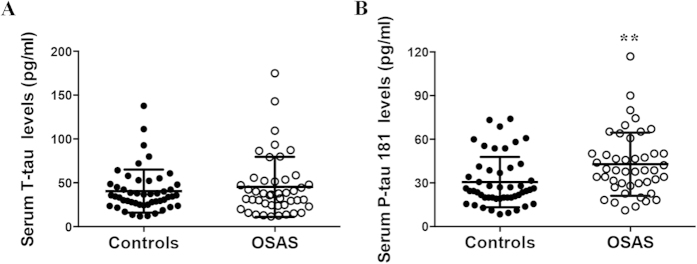
Comparison of the serum tau levels between the controls and patients with OSAS. The serum total tau (**A**) and P-tau 181 (**B**) levels in the controls and patients with OSAS. ^**^denotes *p* < 0.01.

**Figure 5:**
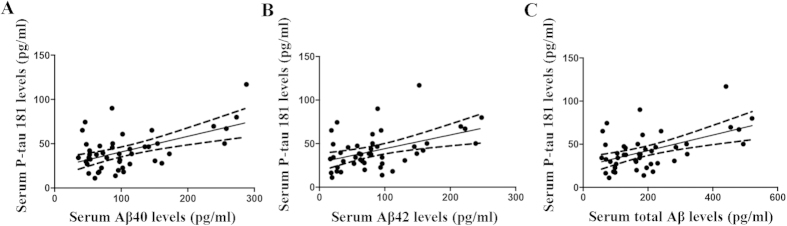
Correlations between the serum Aβ levels and P-tau 181 levels in the patients with OSAS. (**A**) The Aβ40 (r = 0.537, p < 0.001), (**B**) Aβ42 (r = 0.446, p < 0.01) and (**C**) total Aβ (r = 0.511, p < 0.001) levels in the serum were positively correlated with the P-tau 181 levels in the OSAS patients.

To investigate whether neuronal injury is induced in the brains of the OSAS patients, we examined the serum total tau and P-tau 181 levels, both of which are biochemical markers of neuronal injury in the brain^7^,^8^. No differences in the serum total tau levels between the groups was found (45.40 ± 34.24 pg/ml vs. 40.49 ± 24.61 pg/ml, *p* = 0.753), but the serum P-tau 181 levels were strikingly increased in the OSAS patients (42.03 ± 21.58 pg/ml vs. 30.56 ± 17.30 pg/ml, *p* = 0.003; Fig. 4), suggesting that neuronal injuries occurred in the brains of the patients with OSAS. Next, we found that the serum P-tau 181 level was positively correlated with the serum Aβ40 (*r* = 0.537, *p* < 0.001), Aβ42 (*r* = 0.446, *p* < 0.01) as well as total Aβ (*r* = 0.511, *p* < 0.001) levels in the OSAS patients (Fig. 5), suggesting that the OSAS patients with higher Aβ levels may have experienced severe neuronal brain injuries.

## Discussion

To our knowledge, this is the first study to address the serum Aβ and P-tau levels of patients with OSAS. In the present study, we found that compared with the control group, the cognitively normal OSAS patients exhibited significantly higher serum Aβ40, Aβ42 and total Aβ levels, and each of these levels was positively correlated with the severity of OSAS and the extent of hypoxia. Moreover, in the OSAS patients, the serum P-tau 181 levels were higher and correlated with the Aβ levels. These findings suggest that hypoxia might facilitate AD-type pathogenesis in humans.

The causal relationship between OSAS and Aβ levels requires further investigation. Previous studies have indicated that OSAS is associated with increased risks of dementia and AD^9^,^10^. However, patients with Down syndrome, which is associated with an overproduction of Aβ in the brain, exhibited a high prevalence of OSAS^11^, suggesting that higher levels of Aβ might cause OSAS. On the other hand, patients with Down syndrome also exhibit unique upper airway anatomic features and increased risks of obesity, gastroesophageal reflux disease, hypothyroidism, and generalized physical hypotonia^11^. These factors might account for the high prevalence of OSAS among patients with Down syndrome. In OSAS, in addition to nocturnal intermittent hypoxia, poor sleep quality is also an aspect of the pathophysiology of the disease that can impair cerebral metabolism, glucose transport and blood-brain barrier functions and even influence Aβ metabolism in the brain^12^,^13^,^14^. The correlations of serum Aβ and P-tau 181 levels with the severity of OSAS and the extent of nocturnal hypoxia remained after adjusting for the confounders of total sleep time, sleep efficiency and sleep stages, suggesting that hypoxia might increase the levels of Aβ and P-tau in OSAS patients.

Aβ is generated through the sequential proteolysis of the amyloid precursor protein (APP) by the enzymes β-secretase (BACE-1) and γ-secretase, whereas Aβ generation can be avoided via a non-amyloidogenic APP processing pathway that is mediated by α-secretase and γ-secretase^15^. Under conditions of chronic hypoxia, the expression of ADAM10, which is a candidate protein for α-secretase, is decreased in neuronal cells^3^. Additionally, hypoxia increases the BACE-1 level and the enzymatic activity of this protein by enhancing hypoxia-inducible factor 1α (HIF-1-α) expression^16^,^17^,^18^, which results in increased Aβ generation in a mouse model of hypoxia^5^. On the other hand, hypoxia down-regulates the zinc metalloproteinase neprilysin (NEP), one of the most prominent Aβ degrading enzymes^19^. These studies support the perspective that hypoxia may increase Aβ levels by up-regulating its production and down-regulating its degradation in the brain. Aβ levels in the brain and serum form a dynamic equilibrium. The transport of Aβ from the brain into the peripheral blood has been demonstrated in both animal models and humans^20^,^21^, and higher serum Aβ levels might represent higher Aβ burden in the brain. In this regard, our results are consistent with those of previous animal studies that have found that hypoxia is associated with increased Aβ production in the brain^5^,^16^. On another hand, increased serum Aβ levels might also originate from the peripheral organs and tissues that express APP and BACE-1^22^, in response to hypoxia in patients with OSAS. It has been suggested that Aβ originating from the periphery can enter the brain and accelerate AD pathogenesis^23^,^24^. Moreover, elevated Aβ levels in the serum can reduce its clearance from the brain^25^.

Tau is a soluble microtubule-associated protein that is localized in the axons of neuron cells in the brain^26^. Serum phosphorylated tau protein has been suggested to be a biochemical marker of neuronal injury in the brain. In the present study, we found that serum P-tau 181 levels were increased and correlated with the Aβ levels in the OSAS patients. Exposure to hypoxia can induce the hyperphosphorylation of tau in the brains of animals, and this hyperphosphorylation is attributable to the activation of kinases that phosphorylate tau (i.e., glycogen synthase kinase 3 beta, cyclin-dependent kinase 5 and mitogen-activated protein kinase) and the inactivation of kinase that dephosphorylate tau (i.e., phosphorylated protein phosphatase 2A)^27^,^28^. The increased serum levels of phosphorylated tau observed in OSAS patients may result directly from chronic intermittent hypoxia. Moreover, hypoxia can also promote Aβ-induced tau phosphorylation by calpain^29^.

The cerebral blood-oxygen content is essential for neural biosynthetic processes. Hypoxia might induce cerebral hypoperfusion and decrease glucose metabolism, which would disrupt neuronal function and accelerate tau hyperphosphorylation^10^,^30^. Obviously neuronal loss in the hippocampus has been observed in hypoxia-exposed animals^31^. Following hypoxia, reoxygenation could affect blood brain barrier (BBB) function and damage small vessels^10^. Excessive Aβ levels in OSAS could also induce a neurotoxic cascade that leads to neuronal death. Thus, chronic intermittent hypoxia might contribute to neuronal damage and degeneration in the brains of OSAS patients. The increased tau levels observed in the OSAS patients in our study further suggest that repetitive nocturnal hypoxaemia might lead to neuronal degeneration, axonal dysfunction and synaptic loss.

OSAS has been shown to occur more frequently in AD subjects than in cognitively normal older subjects, and its severity correlates with cognitive impairment^32^. Furthermore, OSAS is associated with an increased risk of dementia^9^,^10^. These findings imply that OSAS may promote the development of AD. AD occurs more frequently after the age of sixty, while our OSAS patients were relatively young. Giving that AD-type pathology begins 15–20 years before the onset of dementia in sporadic AD^33^, the elevated Aβ and P-tau levels observed in our patients suggest that OSAS may contribute to the initiation of AD pathogenesis in young patients.

Notably, this study was observational, and we were therefore unable to determine the causal relationship between hypoxia and Aβ. Recent studies have shown that the treatment of severe OSAS with continuous positive airway pressure (CPAP) can slow cognitive decline in patients with AD, which may be due to the correction of intermittent hypoxia^34^,^35^,^36^. However, whether this benefit is associated with decreased Aβ and P-tau levels due to CPAP remains to be investigated. Although we used partial correlations to control for cofounders that included sleep quality, sleep disruption due to chronic fragmentation and restriction may also have affected the results.

In conclusion, this study revealed that increased Aβ levels in the serum are correlated with the severity of chronic intermittent hypoxia in patients with OSAS, and the results thus support the link between the detrimental effects of hypoxia and neurodegeneration, suggesting that OSAS may contribute to the pathogenesis of AD. Because OSAS is a prevalent disease that affects a substantial proportion of the elderly, the treatment of OSAS may have potential to prevent the occurrence and progression of AD.

## Methods

### Study population

A total of 94 subjects with possible OSAS were recruited from the sleep center of the Daping Hospital from September to December of 2014. Among these patients, 45 were ultimately diagnosed with OSAS. Forty-nine age- and gender-matched subjects diagnosed with simple snoring and not OSAS were included as the control group. Subjects were excluded for the following reasons: (1) a family history of dementia; (2) a concomitant neurologic disorder that could potentially affect cognitive function (e.g., severe Parkinson’s disease); (3) severe cardiac, pulmonary, hepatic, or renal diseases or any type of tumour; (4) enduring mental illness (e.g., schizophrenia). Additionally, participants with abnormal cognition according to the Chinese version of the mini-mental state examination (MMSE) were excluded according to protocols have previously been described^37^. The study was conducted in accordance with the Declaration of Helsinki and International Conference on Harmonisation Guidelines for Good Clinical Practice and was approved by Institutional Review Board of Daping Hospital.

### OSAS diagnosis and blood sampling

The diagnosis of OSAS was based on the daytime and nocturnal symptoms and nighttime polysomnography (PSG)^38^, which included records of electroencephalography (EEG), electrooculography (EOG), submental and bilateral tibial muscle electromyography (EMG), electrocardiography (ECG), oxygen saturation, oral and nasal airflow and respiratory effort. TST refers to the time from sleep onset to the end of the final sleep epoch minus the time awake. Sleep efficiency was defined as the ratio of the TST to the time in bed. AHI was defined as the total number of complete cessations (apnea) and partial obstructions (hypopnea) of breathing that occurred by per hour of sleep. Arousal was defined as an interruption of sleep lasting more than 3 seconds. The ArI referred to the total number of arousals divided by the number of hours of sleep. ODI was defined as the number of times per hour of sleep that the blood oxygen level dropped by 3 per cent or more relative to baseline. Based on the AHI, the severity of OSAS was classified as follows: mild OSAS (5 ≤ AHI < 15), moderate OSAS (15 ≤ AHI ≤ 30), or severe OSAS (AHI > 30)^38^. Demographic data including age, gender, height, weight and educational level, were collected upon admission. BMI was calculated by dividing the weight (kg) by the square of the height (m). Medical histories were collected as per our previous study from the medical records and a formal questionnaire that included current medications^37^. The data included prior head trauma and surgery, prior gas poisoning, schizophrenia, hypothyroidism, coronary heart diseases, atrial fibrillation, cerebrovascular diseases, chronic obstructive pulmonary disease, chronic hepatitis, chronic renal insufficiency, hypertension, diabetes mellitus, hypercholesterolemia, Parkinson’s disease, and regular use of non-steroidal anti-inflammatory and prescription drugs. Fasting blood was sampled between 06:00 and 07:00 to avoid the variation related to possible circadian rhythm effects. The blood samples were centrifuged immediately after drawn and then stored at −80 °C until use. Informed consent was obtained from each participant before the acquisition of the blood sample.

### Measurements of serum Aβ and tau levels

Serum Aβ40 and Aβ42 levels were determined using human Aβ enzyme-linked immunosorbent assay (ELISA) kits (Covance, Princeton, NJ, USA). Serum total tau and P-tau 181 levels were detected using human tau ELISA kits (Invitrogen, Carlsbad, CA, USA). All of the ELISA measurements were performed according to the manufacturers’ instructions. The samples and standards were measured in duplicate, and the means of the duplicates were used for the statistical analyses.

### Statistical analysis

The differences in demographic characteristics and serum Aβ levels between the groups were assessed with two-tailed independent t-tests, Mann-Whitney U tests or Chi-squared tests as appropriate. Partial correlation analyses were used to investigate the associations of serum Aβ levels with the AHI, ODI, MSaO_2_, and LSaO_2_ values. Spearman correlation analyses were used to examine the correlations between the serum Aβ levels and P-tau 181 levels. The data are expressed as the mean ± standard deviation (SD). All hypothesis testing was two-sided, and *p* < 0.05 was defined as statistically significant. The computations were performed with SPSS version 19.0 (SPSS Inc., Chicago, IL, USA).

## Additional Information

**How to cite this article**: Bu, X.-L. *et al.* Serum amyloid-beta levels are increased in patients with obstructive sleep apnea syndrome. *Sci. Rep.* **5**, 13917; doi: 10.1038/srep13917 (2015).

## Supplementary Material

Supplementary Table 1

## Figures and Tables

**Table 1:** Characteristics of the study population.

	Controls (n = 49)	OSAS (n = 45)	P value
Age, years	42.98(9.62)	44.31(9.96)	0.511
Gender, male, n (%)	34(69.39)	33(73.33)	0.82
Education, years	14.27(3.46)	13.87(3.79)	0.595
Current smokers, n (%)	20(40.82)	17(37.8)	0.834
BMI, kg/m^2^	26.07(2.61)	26.84(2.52)	0.146
Hypertension, n (%)	6(12.24)	7(15.56)	0.768
Diabetes mellitus, n (%)	2(4.08)	1(2.22)	1
Cardiovascular disease, n (%)	2(4.08)	3(6.67)	0.668
Hyperlipidemia, n (%)	7(14.29)	5(11.11)	0.761
Total sleep time, min	404.91(51.67)	397.30(43.84)	0.461
Sleep efficiency, %	85.24(7.32)	84.56(9.86)	0.892
AHI, events/h	2.26(1.35)	32.68(23.34)	<0.001
ArI, events/h	8.83(4.21)	32.62(16.75)	<0.001
Sleep stages 1 and 2, %	56.76(10.53)	62.86(15.31)	0.003
Sleep stage 3, %	25.48(10.33)	23.19(17.72)	0.030
REM, %	17.76(5.99)	13.95(6.86)	0.006
Mean SaO_2_, %	95.61(1.17)	90.93(2.90)	<0.001
Lowest SaO_2_, %	91.20(1.68)	80.60(7.17)	<0.001
ODI, events/h	2.17(1.27)	26.45(17.76)	<0.001

Data given as Mean ± SD unless otherwise indicated. Abbreviations: AHI, apnea-hypopnea index; ArI, arousal index; BMI, body mass index; ODI, oxygen desaturation index; REM, rapid eye movement; SaO_2_, oxyhaemoglobin saturation. *P* value, two-tailed independent t-tests, Mann-Whitney U tests or chi-squared tests as appropriate.
