# Supplementary material for: Serum amyloid-beta levels are increased in patients with obstructive sleep apnea syndrome
Source: Sci Rep. 2015 Sep 9;5:13917. doi: 10.1038/srep13917 (PMC4563592; doi:10.1038/srep13917)
Supplement: Supplementary Table 1 [file srep13917-s1.pdf]

Serum Amyloid-Beta Levels Are Increased In Patients With Obstructive Sleep Apnoea Syndrome

Xian-Le Bu[1,*]; Yu-Hui Liu[1,*]; Qing-Hua Wang[1]; Shu-Sheng Jiao[1]; Fan Zeng[1]; Xiu-Qing Yao[1]; Dong Gao[2]; Ji-Chuan Chen[3]; Yan-Jiang Wang[1]

[1]Department of Neurology and Center for Clinical Neuroscience, [2]Department of Sleep center, [3]Department of Otolaryngology Head and Neck Surgery, Daping Hospital, Third Military Medical University, 10 Changjiang Branch Road, Yuzhong District, Chongqing 400042, China

[*] These authors contributed equally to this work

Correspondence to: Yan-Jiang Wang, email: yanjiang_wang@tmmu.edu.cn,

Telephone: +86 23 68757850, facsimile: +86 23 68711956

Supplementary tables

Supplementary Table 1 The correlations of serum Aβ levels with AHI, ODI, mean SaO_2 and lowest SaO_2 in all participants.

	Aβ40		Aβ42		Total Aβ	
	γ	P	γ	P	γ	P
AHI, events/h	0.301	0.004	0.306	0.003	0.315	0.002
ODI, events/h	0.449	<0.001	0.489	<0.001	0.485	<0.001
Mean SaO_2	-0.261	0.023	-0.249	0.03	-0.256	0.026
Lowest SaO_2	-0.231	0.044	-0.234	0.042	-0.227	0.049

Partial correlation analysis, adjusted for age, gender, education, smoking, BMI and comorbidities. Abbreviations: AHI, apnoea-hypopnoea index; ODI, oxygen desaturation index; SaO_2, oxyhaemoglobin.
